# Preliminary Investigation of a Novel Measure of Speech Recognition in Noise

**DOI:** 10.3390/audiolres15030059

**Published:** 2025-05-13

**Authors:** Linda Thibodeau, Emma Freeman, Kristin Kronenberger, Emily Suarez, Hyun-Woong Kim, Shuang Qi, Yune Sang Lee

**Affiliations:** Department of Speech, Language, and Hearing, School of Behavioral and Brain Sciences, University of Texas at Dallas, Richardson, TX 75080, USA

**Keywords:** speech recognition, hearing tests, signal-to-noise ratio, syntactic complexity, auditory training, aural rehabilitation

## Abstract

**Background/Objectives:** Previous research has shown that listeners may use acoustic cues for speech processing that are perceived during brief segments in the noise when there is an optimal signal-to-noise ratio (SNR). This “glimpsing” effect requires higher cognitive skills than the speech tasks used in typical audiometric evaluations. Purpose: The aim of this study was to investigate the use of an online test of speech processing in noise in listeners with typical hearing sensitivity (TH, defined as thresholds ≤ 25 dB HL) who were asked to determine the gender of the subject in sentences that were presented in increasing levels of continuous and interrupted noise. **Methods:** This was a repeated-measures design with three factors (SNR, noise type, and syntactic complexity). Study Sample: Participants with self-reported TH (N = 153, ages 18–39 years, mean age = 20.7 years) who passed an online hearing screening were invited to complete an online questionnaire. Data Collection and Analysis: Participants completed a sentence recognition task under four SNRs (−6, −9, −12, and −15 dB), two syntactic complexity settings (subjective-relative and objective-relative center-embedded), and two noise types (interrupted and continuous). They were asked to listen to 64 sentences through their own headphones/earphones that were presented in an online format at a user-selected comfortable listening level. Their task was to identify the gender of the person performing the action in each sentence. **Results:** Significant main effects of all three factors as well as the SNR by noise-type two-way interaction were identified (*p* < 0.05). This interaction indicated that the effect of SNR on sentence comprehension was more pronounced in the continuous noise compared to the interrupted noise condition. **Conclusions:** Listeners with self-reported TH benefited from the glimpsing effect in the interrupted noise even under low SNRs (i.e., −15 dB). The evaluation of glimpsing may be a sensitive measure of auditory processing beyond the traditional word recognition used in clinical evaluations in persons who report hearing challenges and may hold promise for the development of auditory training programs.

## 1. Introduction

In noisy environments, such as restaurants, a crowded room, or an elementary classroom, many factors may negatively impact speech perception. Physical room characteristics may include detrimental effects of reverberation caused by high ceilings and hard floors. Multiple speakers talking simultaneously increases background noise or sudden loud impulse noises can occur in the surrounding environment. These characteristics decrease the signal-to-noise ratio (SNR), making the speaker’s voice less intense than the background noise. A more challenging SNR makes it difficult to communicate and hampers speech perception in those with typical hearing (TH, defined as thresholds ≤ 25 dB HL) and can make it more difficult for those with hearing loss [1]. It is known that speech recognition improves as the SNR increases [2]. However, in the traditional clinical audiological evaluation, speech recognition testing is performed in quiet and using single words that listeners are asked to repeat. Furthermore, in the real world, background noise is often fluctuating, which may allow listeners to receive useful cues during the weaker components of the competition. These cues may be held in short-term memory and combined over time to determine meaning. This skill has been referred to as “glimpsing” and has not been included as part of routine clinical audiological assessments.

Glimpsing is a technique that allows an individual to gain meaning from a speech signal by utilizing speech cues that occur in the silent periods or gaps within the noise. When listening in challenging noisy environments such as a crowded restaurant, persons typically use glimpsing when the nearby competing conversation periodically subsides in order to hear the acoustic cues from their communication partners. As the glimpsing process continues, persons will combine the fragments received in the gaps in noise to gain meaning. This process was first described by Miller and Licklider in 1950, where they determined whether interrupting acoustic speech signal in different manners influenced the perception for the listener. It was concluded that in regularly spaced bursts of noise, the higher the SNR, the higher the accuracy of recognizing speech in the presence of background noise [3]. This foundational work also indicated a negative relationship between speech recognition and the frequency of interruptions. When the interruptions occurred with greater frequency, the gaps of silence became smaller, and speech recognition declined. Many studies have since been conducted to determine if glimpsing is an effective way to improve speech recognition scores. Cooke [4] conducted a study of 12 different conditions with various SNRs from −7 to +8 dB, frequency modulations, and amplitude modulations and demonstrated the proportion of glimpsing could work as a good predictor of intelligibility. It was suggested that the best fit model could predict behavioral data when regions in the acoustic signal with a local SNR > −5 dB were considered potential opportunities for glimpsing. Evaluation of the ability to benefit from glimpsing at even more demanding SNRs (−6, −9, −12, and −15 dB) would contribute to our understanding of this effect in listeners with TH.

This ability was studied in persons with hearing loss by Fogerty et al. [5] who showed differences in speech recognition scores in the presence of background noise depending on whether the noise was continuous (i.e., being played during the entire duration of the speech signal) or interrupted (i.e., glimpsing). The interrupted signal contained gaps in the noise that allowed parts of the speech signal to be perceived in the absence of noise. Consistently poorer speech recognition performance was reported in older listeners with hearing loss compared to those age-matched peers with TH. They proposed that a decline might be related to impaired temporal processing abilities.

In addition, “glimpsing” is reported as a skill that can be improved as a learning effect [6,7]. Specifically, Rhebergen et al. [7] found the speech reception threshold improved by about 3–4 dB SNR after five replications in interrupted noise relative to the initial threshold. Sullivan et al. [8] also reported improvements in glimpsing following three months of auditory training in interrupted noise compared to training in continuous noise in 24 children with moderate-to-severe hearing loss. Similar speech recognition improvement was found for those trained in interrupted noise when tested in both interrupted and continuous noise conditions while only modest improvements existed under interrupted noise tests for those trained in continuous noise. These findings confirmed the benefits of auditory training under interrupted noise on speech recognition in noise for children with hearing loss. It was suggested that glimpsing was a skill that could be improved through training and may serve as a promising clinical application in auditory rehabilitation.

The ability to use acoustic speech cues available during silent intervals may lead to more sensitive measures of auditory processing. Such results could ultimately result in beneficial training protocols. Such training might involve practicing speech recognition of sentences presented simultaneously with interrupted noise in which the gaps become progressively smaller. Currently, speech-in-noise (SIN) tests are typically performed in continuous background noise and are thought to reflect the individual’s capability in the real world [9,10]. However, there has been limited attention paid to audiometric tests that assess auditory processing beyond mere recognition of a stream of words at the sentence level. Hearing loss is indeed more vulnerable to spoken sentences with a more complex syntactic structure, suggesting interactions between acoustic processing and higher-order cognitive operations [11,12].

In contrast to using word repetition, Lee et al. [13] used a task that required a rapid analysis of syntactic relations within a sentence. The stimuli were always spoken by a male, but the complexity of sentence stimuli was manipulated by using either subject-relative or object-relative sentences. In a sentence with both a subject and an object, sentence relative (SR) clauses involve the action being performed by the subject, i.e., “Girls that comfort dads are kind”. In contrast, object relative (OR) clauses are object-centered, with the action being performed by the object, resulting in higher syntactic complexity, i.e., “Dads that girls comfort are kind”. For each sentence, the listeners were instructed to indicate whether a male or a female performed the action. This paradigm has been used to demonstrate a robust syntactic effect with a higher-level of difficulty for the OR than the SR sentences. This is presumably due to the increasing working memory load and/or temporal complexity [14,15,16,17,18].

Using these syntactically complex sentences in conditions where glimpsing is possible (i.e., interrupted noise) compared to conditions where it is not possible may provide insights into the difficulties that people experience in real-world noisy conditions where they must not only recognize the speech but also cognitively process what they hear. It is expected that as SNR increases from negative to positive values, more acoustic information is received. In addition, if there are gaps in the noise, additional cues may be received which will lead to greater accuracy. By using the cognitively demanding task involving varying sentence structure, additional skills such as short-term memory and syntactic knowledge can be assessed and may more accurately reflect the real-world communication difficulties.

The aim of this study was to evaluate a novel, auditory speech perception-in-noise test administered online in adults with TH. This test consisted of the SR/OR sentences played in continuous/interrupted noise under four SNRs and required the participant to process the syntax to determine if the action was performed by a male or female. This task involved the retention of important information from each sentence, which is a higher-level task targeting working memory than simple sentence repetition. In addition, the cognitive load increased as the listening became more difficult at the lower SNRs, in the continuous versus interrupted noise, and as the sentence type changed from SR to OR. It is hypothesized that listeners with TH would perform better with higher SNRs and when listening in the interrupted versus continuous noise conditions. Furthermore, listeners might benefit more from the “glimpsing” as shown by the gap between the two noise conditions as the SNR decreased.

## 2. Materials and Methods

### 2.1. Participants

A total of 158 participants were involved in this study which agreed with the estimated sample size needed to obtain a medium-effect size with an error probability of 0.05 [19]. Five responses were deleted due to incomplete information. Responses included 153 adults, aged 18–39 years (mean = 20.7 years). Participants were recruited through a university research participant-recruiting platform and email distribution to various student groups. Informed consent was obtained by each participant at the beginning of the survey which was approved by the Institutional Review Board (IRB). All participants had to self-report TH bilaterally as determined with an online hearing screening and complete all test questions. Exclusion criteria included self-reported hearing loss or identified through a hearing screener or inability to complete this study due to unforeseen circumstances and technical difficulties.

### 2.2. Stimuli

The stimuli were taken from those developed by Lee et al. [13] and included a total of 80 sentences (the first 16 were practice). Stimuli from the Lee et al. [13] study were mixed with babble noise using Adobe Audition and uploaded to Qualtrics as MP3 files. There were three difficulty variables which included SNR, glimpsing (interrupted vs. continuous noise), and sentence type (SR vs. OR). An example of an SR sentence is “Daughters that charm fathers are joyful” and an example of an OR sentence is “Boys that women scold are mean”. The sentences had been normalized by root-mean-square values (RMS) to minimize the use of intensity variations as perceptual cues. The following four sentence structures were used: OR spoken by a male or female and SR spoken by a male or female.

To familiarize the participants with the task, a practice set of 16 sentences was presented in quiet and at easy SNRs (0 and +3 dB SNR). Eight sentences were played in continuous and eight in interrupted noise with SR/OR type evenly distributed. The actual test session consisted of four SNRs at −6, −9, −12, and −15 dB, with 16 sentences played in each SNR. Among these 16 sentences, 8 were presented in continuous noise (4 SR and 4 OR) and 8 were presented in interrupted noise (4 SR and 4 OR). The stimuli (SNR, noise, and sentence type) were randomized within this study using a random number generator.

Multi-talker babble noise was used for the background noise stimuli in both continuous and interrupted conditions [20]. Using Adobe Audition for audio editing, the original noise file was first decreased in amplitude to avoid peak clipping and was matched to the RMS of the sentences to create the 0 dB SNR condition. The background noise, either continuous or interrupted, was increased systematically and mixed with the sentence stimuli at a steady intensity to create the four SNRs. To avoid identical noise samples mixed with the sentences, a random section of noise was selected to mix with each sentence such that there was one second of noise before and after the sentence. The interrupted noise files were made by taking the noise file and randomly segmented it by sections of 20, 50, 75, or 95 ms of silence to create silent gaps. Sentences were mixed with the interrupted noise files in the same way as for the continuous noise. When the interrupted noise files were played, it created a “choppy” effect that allowed the listener to hear parts of the sentence through the noise.

### 2.3. Procedure

**Figure 1:**
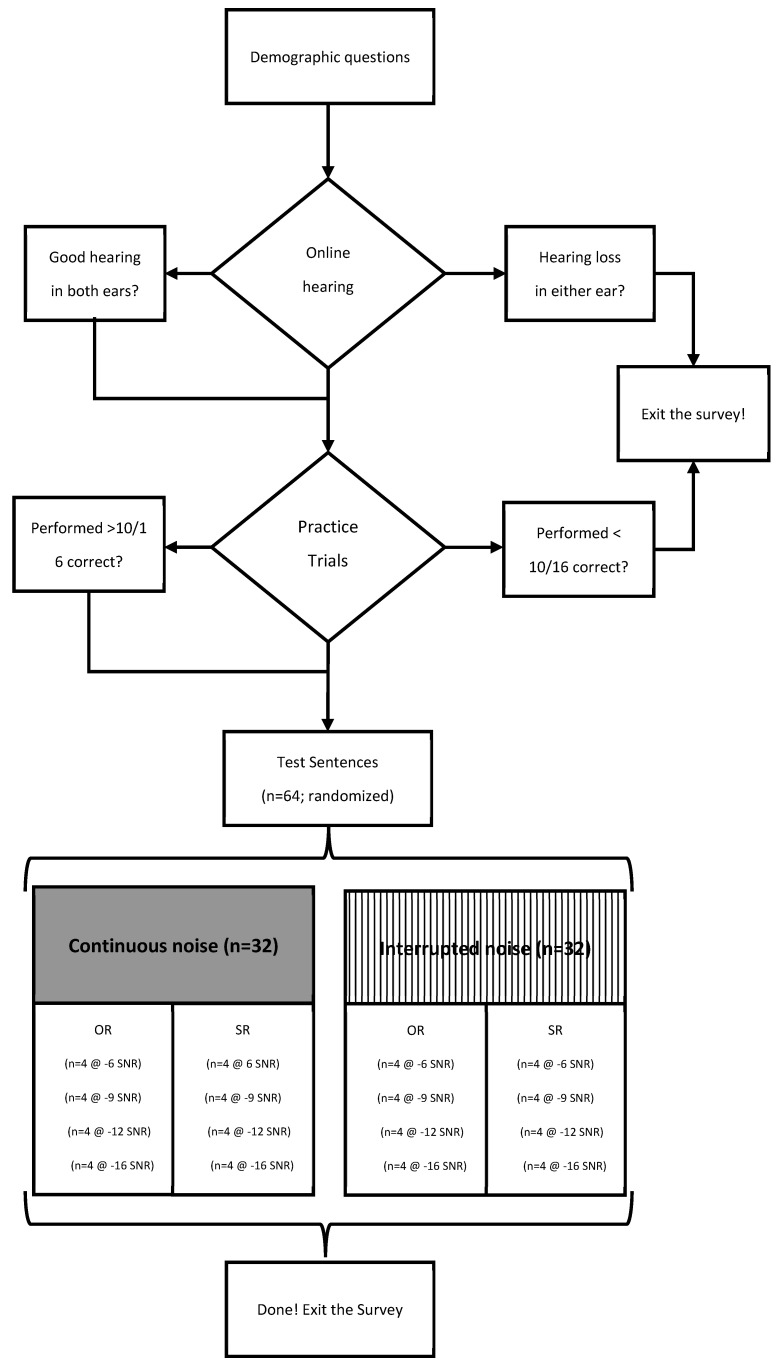
Sequence of online survey. Note: SNR = signal-to-noise ratio; SR = subject-relative; OR = object-relative.

Anonymous responses were collected through the online web-based platform Qualtrics supported by the University of Texas at Dallas in the order shown in Figure 1. Participants were allowed to complete the survey at their home or in any quiet location with minimal interruptions. They were asked to use wired or wireless stereo headphones or earphones. After completing the consent and basic demographic information, the participants were redirected by a link to the online hearing screener ShoeboxOnline/Signia (https://www.signia.net/en-us/service/hearing-test/ (accessed on 1 September 2023)). Following some questions regarding general hearing abilities, the user was asked to use headphones (wired or wireless) and to adjust the volume of the computer to max level. First, continuous speech was presented followed by tones with each ear tested separately. The user adjusted the volume buttons on the screen to the highest comfortable level and the lowest level that speech could be understood. The final step was the volume adjustment for barely audible warbled tones. The screener would record a hearing level as “normal”, “loss”, or “severe loss”. Participants were instructed to discontinue this study if they reported their hearing acuity as a “loss” or “severe loss” for either ear.

Prior to listening to the stimuli, participants were asked to play a single clip of multi-talker babble and adjust the volume of their headphones/earphones to a comfortable listening level using the volume slider on the device they were using. They were instructed to leave their volume here for the remainder of this study. In total, this study took approximately 1 h to complete. After the volume was adjusted, a practice section was completed first to ensure the participants understood the task and timing of the questions. Correct answer feedback was provided during these 16 questions. Participants were instructed to remain in a quiet environment without interruptions; if this was not possible for the length of the entire task, they were instructed to please exit and try again at another time. Each screen was timed and would automatically advance to the next question after 10 s to prevent the participants from playing the sentence more than once. The practice questions were also timed to allow the practice of both listening to the stimuli and selecting the answer choice they heard in the appropriate amount of time. Participants were required to meet the practice criteria of 10/16 questions correctly answered to advance to the main study questions. Because the task of identifying the subject is not as common as repeating words, performance at 63% accuracy was selected as an indication that participants understood and could attend to the task.

The practice and main survey questions took about one hour to complete and involved listening to the sentences spoken by a male with particular attention to who was performing the action, a male or female. The instructions were “Please play the following clip and identify the gender of the people performing the action: male or female”. For example, the answer to this OR sentence, “Girls that dads comfort are kind”, was male, as the dads are performing the action (comfort). Each sentence was made into both OR and SR syntax. For example, the above OR sentence was also presented in SR form as “Girls that comfort dads are kind” with a female conducting the action in this sentence. This required cognitive processing and working memory as the participants did not know which sentence structure they would receive each time.

Four SR and four OR sentences were used per SNR level for continuous noise and also for interrupted noise. These stimuli were randomly placed within the Qualtrics study. Participants were given ten seconds to answer the survey questions before the page auto-advanced. If a question was not answered in the allotted time, then the trial was scored as incorrect. Questions were automatically scored into categories based on four SNR levels (−6, −9, −12, −15), two noise types (continuous and interrupted), and two sentence types (SR and OR).

### 2.4. Data Analysis

The accuracy of individual trials was entered into a mixed-effect logistic regression model using the glmer function of the lme4 package [21], to analyze participants’ comprehension performance. The model included sentence type (SR and OR), noise type (continuous and interrupted), SNR (−6, −9, −12, and −15 dB), the respective two-way and three-way interactions as fixed factors, and item and participant as random intercepts. Effect sizes were determined according to Cohen’s *d* values.

## 3. Results

**Figure 2:**
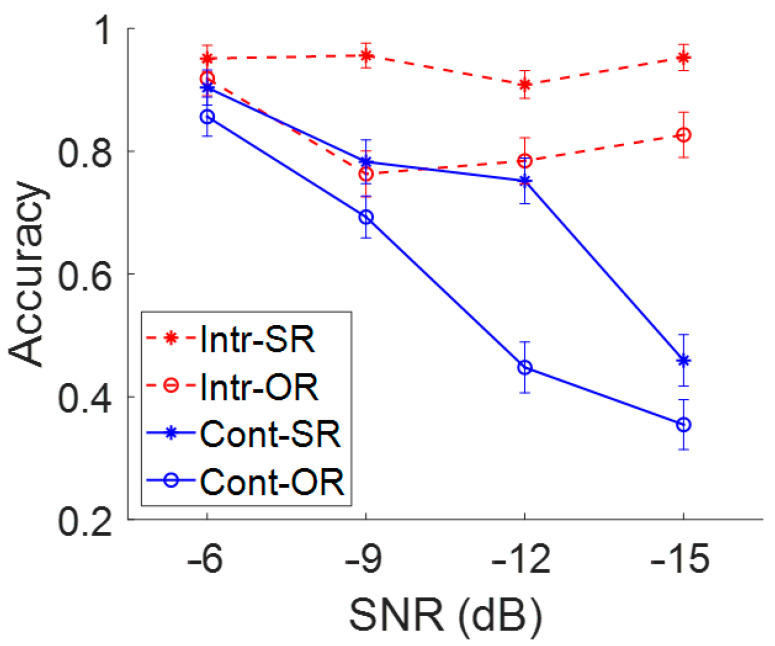
Average speech recognition accuracy across sentence type, noise type, and signal-to-noise ratio. Note: SNR = signal-to-noise ratio; Intr = interrupted noise; Cont = continuous noise; SR = subject-relative; OR = object-relative.

To examine the results of the online measurement of speech recognition in noise, the performance for the two sentence types (SR vs. OR) presented in two types of noise (interrupted and continuous) at four SNR levels (−6, −9, −12, −15 dB) was reviewed. As expected, all three main effects were significant. Interactions were non-significant except for the noise type by SNR. The group-averaged accuracy for each combination of stimulus conditions is shown in Figure 2 and Table 1. The main effect of sentence type was significant (*b* = 0.50, *SE* = 0.09, *z* = 5.53, *p* < 0.001, small Cohen’s *d* = 0.47), indicating lower accuracy for the OR than SR sentences. We also found the main effects of noise type (*b* = 0.80, *SE* = 0.09, *z* = 8.74, *p* < 0.001, large Cohen’s *d* = 0.90) and SNR (*b* = 0.51, *SE* = 0.08, *z* = 6.21, *p* < 0.001), indicating that participants performed worse with a lower SNR and when the noise was continuous compared to interrupted. Importantly, the noise type significantly interacted with the SNR factor (*b* = −0.37, *SE* = 0.08, *z* = −4.57, *p* < 0.001), indicating that the effect of SNR on sentence comprehension was more pronounced in the continuous noise compared to the interrupted noise condition (Figure 2). However, the two-way interactions including the sentence type as well as the three-way interaction were not significant (sentence type × noise type: *b* = −0.14, *SE* = 0.09, *z* = 1.55, *p* = 0.121, sentence type × SNR: *b* = −0.07, *SE* = 0.08, *z* = −0.82, *p* = 0.414, and sentence type × noise type × SNR: *b* = −0.02, *SE* = 0.08, *z* = −0.23, *p* = 0.818). The non-significant two-way interactions with sentence type were not surprising given the previous research showing the greater difficulty with OR relative to SR sentences. With this consistent pattern of OR performance lower than SR performance, the lack of a significant three-way interaction was also not surprising.

## 4. Discussion

In this study, the key findings were the significant main effects of SNR, the noise type, as well as their two-way interactions. As shown in Figure 2, poorer recognition accuracy was identified with a lower SNR setting, but only when tested with continuous noise. The accuracy score remained high across the four SNRs when tested in interrupted noise. The significant impact of SNR level found in this study was in accordance with the previous studies [9,10] as well as the glimpsing effect as indicated by the noise type [4]. This study extended the range of glimpsing effect to a lower SNR (i.e., −15 dB). The significant SNR by noise-type two-way interaction indicated the benefits of the glimpsing effect despite adverse listening conditions. Specifically, even under a demanding listening condition of −15 dB SNR, the performance remained over 80%. Relative to the decreased accuracy with lower SNRs, when tested with continuous noise, the benefit of a glimpsing effect increased as the SNR decreased. This suggests that as the perceptual task becomes more difficult, persons with TH can rely more on the cues they receive during the gaps in the interrupted noise.

Some implications of these findings include the effects of the challenging listening task on the individual such as increased listening effort and the sensitivity of this task to reflect differences in auditory processing. Our results suggested the need to include listening effort measures (i.e., cognitive demands when completing a listening task) in future investigations. In a pilot study conducted in the lab setting, the same stimuli were tested by another 11 participants with TH and all participants were required to report their subject listening effort using the NASA-Task-Load-Index [22] after each noise type. Across the four SNRs and two clause structures, listeners reported lower listening effort (52.61%) when tested in interrupted noise relative to continuous noise (70.70%). Although this testing was conducted in the lab, the pilot data imply potentially reduced listening effort by the glimpsing effect.

The current study also showed a significant impact of syntactic complexity on individual auditory processing performance. As expected, participants had greater accuracy when recognizing the SR-structured sentences compared to OR-structured sentences. Although the perceptual difficulty was set differently (noise type/SNR vs. speech rate/aging/hearing loss), our findings were parallel to Wingfield et al. [12]. The performance for the easy conditions in the current study (i.e., interrupted noise/high SNR) was similar to the performance in the Wingfield et al. [12] study for the slower rate condition by the younger group/typical hearing. Likewise, the performance in the hard condition in the current study (i.e., continuous noise/lower SNR) was similar to that in the Wingfield et al. study [12] for the fast-rate condition by the older group/hearing loss. This suggests that the SR/OR stimuli may be a sensitive measure of differences in auditory processing. There may be additional factors that influence performance such as linguistic proficiency, working memory, or syntactic familiarity. Future studies could include additional measures of these abilities to determine the contributions of each that should be considered when determining rehabilitative programs and/or technology. If there is a significant contribution of working memory to the task performance, training to improve that skill may be considered. However, if linguistic proficiency showed a stronger contribution, then training may focus more on that ability. In either case, the use of remote microphone technology may provide a more immediate benefit to persons with HD despite TH.

The greatest limitations of this study relate to the lack of control during online testing. It is expected with online audio testing that variability will naturally occur with the use of various headphones and allowing the user to adjust their volume settings. To determine the range of these variations, a pilot study was conducted. Using the digits-in-noise task at the same SNR values, there was no significant difference when the online stimuli were presented in the lab with one set of headphones versus through individual computers at home with a variety of headphones. Though the home setting could provide the participants with a more comfortable testing environment, it could undermine the data quality that may occur with uncontrolled transducers (headphones, earphones), intensity (selected volume levels were not reported), extraneous environmental sounds, etc. As part of the pilot study, the online screening measure was also compared to traditional audiometric testing in a sound booth and there was agreement for 91% of the participants. A final limitation is that the sensitivity of the measures was constrained by the high probability of guessing the correct answer because there was only two choices (male/female).

These findings might be beneficial in future clinical applications. It is possible that some training in temporal processing could result in rehabilitation benefits for those with hearing loss who have poor temporal processing. For example, Weissgerber and Baumann [23] reported an 8.7 dB decrease in the speech reception threshold using amplitude-modulated masking noise (i.e., “Fastl-noise”) relative to continuous broadband noise (i.e., “Olnoise”). This training effect might be dependent on residual hearing levels. Several researchers have reported an absence of the glimpsing effect in bilateral cochlear implant users or subjects using electric-acoustic stimulation (i.e., vocoded speech) [23,24,25]; meanwhile, others have reported the potential glimpsing effect may benefit bimodal listeners [24]. This suggests the need to fill a gap in the literature regarding the role of temporal processing and speech recognition in noise. Examination of the findings in groups with different degrees of residual hearing may provide insights into determining candidacy for temporal processing training.

Sullivan et al. [8] confirmed the potential benefits of a three-month auditory training program under interrupted noise of 0-, 6-, and 12-dB SNR on SIN recognition for children with hearing loss. A 1 dB improvement on the HINT is equivalent to improvements in speech intelligibility by 8.9% [26]. Sullivan et al. [8] reported an average post-training improvement of 7.46 and 7.13 dB when evaluated in interrupted and continuous noise, respectively. This corresponds to a predicted 63% recognition improvement based on the 1 dB to 8.9% transformation. However, both the training and test conditions were at SNRs > 0 dB, which do not reflect the common negative SNR conditions present in daily life. The current findings suggest the extension of the glimpsing benefit to a lower SNR although testing was conducted with listeners with TH. Working memory training may also be an effective way of improving speech perception in noise [27]. Following the rationale of Sullivan et al. [8], such training may be more effective in interrupted noise compared to continuous noise. When taken together, the results suggest possible promising benefits of auditory training in interrupted noise in lower SNR settings. Similarly, the parallel findings with Wingfield et al. [12] also suggest that the perceptual difficulty is impacted by other factors including aging and speech rate.

Future research may focus on the exploration of the combination of factors including noise type and syntactic complexity for auditory training in clinical rehabilitation programs. Compared to control groups with no auditory training to improve glimpsing skills, individuals who enroll in training programs that are designed to increase working auditory skills during specifically designed noise may show significantly better speech recognition in noise. The training hierarchy might begin with monosyllabic words spaced exactly during the gaps in the background noise and progress to conditions with greater word length and overlapping intermittent noise. The ultimate goal would be to enhance speech recognition in more naturalistic situations such as increasing one’s ability to follow a story presented in typical multi-talker babble akin to that encountered in a restaurant. Following the conclusive evidence of the training benefits, the application of training protocols could be implemented by a manufacturer into the corresponding hearing aid application on a smartphone. The application would include a sequence of training levels that would be presented to the user via the personal hearing technology and scored based on responses spoken by the user into the smartphone.

## 5. Conclusions

Individuals may encounter communication deficits when listening with background noise despite TH sensitivity. To explore the impact of different factors on individual auditory processing performance in noise, 153 listeners with TH were tested under three perceptual challenges (SNR level, sentence syntax complexity, and noise type). Significant main effects of each difficulty factor as well as the SNR by noise-type two-way interaction were found in the regression model, while the other two-way interactions and three-way interactions were nonsignificant. Auditory processing skills involve higher-level abilities beyond the simple repetition of what was heard especially under adverse listening conditions. Listeners with TH show benefit from the glimpsing effect even under low SNRs (i.e., −15 dB). Evaluation of one’s ability to use glimpsing with syntactically complex sentences may lead to more sensitive clinical measures of speech processing beyond the typical task of simple repetition. It could be that two persons with the same degree of hearing loss might perform very differently on a task involving glimpsing. Those individuals who score the same on speech processing tasks presented in continuous versus interrupted noise (i.e., limited glimpsing) may be candidates for auditory training. Such training may focus on progressively complex tasks that vary in gap duration in the competing noise, memory load of the sentence length, and/or syntactic complexity. However, further research is needed to establish a paradigm involving glimpsing that may be used as a clinical measure of auditory processing and possible training program in auditory rehabilitation.

## Figures and Tables

**Table 1:** Average speech recognition accuracy across the noise and sentence-type conditions as a function of SNR.

	Continuous		Interrupted	
SNR (dB)	SR	OR	SR	OR
−6	0.90	0.86	0.95	0.92
−9	0.78	0.69	0.96	0.76
−12	0.75	0.45	0.91	0.78
−15	0.46	0.35	0.95	0.83

Note: SNR = signal-to-noise ratio.

## Data Availability

The data presented in this study are available on request from the corresponding author due to privacy concern.

## Abbreviations

The following abbreviations are used in this manuscript:

dB	Decibel
OR	Object relative
SR	Subject relative
TH	Typical Hearing
